# Psychophysiological Indicators for Modeling User Experience in Interactive Digital Entertainment †

**DOI:** 10.3390/s19050989

**Published:** 2019-02-26

**Authors:** Martin Čertický, Michal Čertický, Peter Sinčák, Gergely Magyar, Ján Vaščák, Filippo Cavallo

**Affiliations:** 1 Department of Cybernetics and Artificial Intelligence, Technical University in Košice, Letná 9, 040 01 Košice, Slovakia; peter.sincak@tuke.sk (P.S.); gergely.magyar@tuke.sk (G.M.); jan.vascak@tuke.sk (J.V.); 2 Department of Computer Science, Czech Technical University in Prague, 166 36 Prague, Czech Republic; certicky@gmail.com; 3 The Biorobotics Institute, Scuola Superiore Sant’Anna, 560 25 Pisa, Italy; filippo.cavallo@santannapisa.it

**Keywords:** machine learning, psychophysiological measures, user experience, modeling, digital entertainment, enjoyment, heart rate, respiratory activity, electroencephalography, galvanic skin response

## Abstract

Analyses of user experience in the electronic entertainment industry currently rely on self-reporting methods, such as surveys, ratings, focus group interviews, etc. We argue that self-reporting alone carries inherent problems—mainly the misinterpretation and temporal delay during longer experiments—and therefore, should not be used as a sole metric. To tackle this problem, we propose the possibility of modeling consumer experience using psychophysiological measures and demonstrate how such models can be trained using machine learning methods. We use a machine learning approach to model user experience using real-time data produced by the autonomic nervous system and involuntary psychophysiological responses. Multiple psychophysiological measures, such as heart rate, electrodermal activity, and respiratory activity, have been used in combination with self-reporting to prepare training sets for machine learning algorithms. The training data was collected from 31 participants during hour-long experiment sessions, where they played multiple video-games. Afterwards, we trained and compared the results of four different machine learning models, out of which the best one produced ∼96% accuracy. The results suggest that psychophysiological measures can indeed be used to assess the enjoyment of digital entertainment consumers.

## 1. Introduction

The electronic entertainment (EE) industry has undergone extensive growth over the past decade. In the USA alone (a country with the biggest video-game market in the world), sales went from $7.3 billion in 2006 to $30.4 billion spent in the game industry in 2016. The global value of the video-game industry is estimated to reach $138.4 billion by 2021 [1]. Yet, the assessment of user experience in video-games is still primarily done using self-report techniques, such as questionnaires and interviews [2].

We believe that this field may benefit from progress made in machine learning, especially with an increased number of game developers taking advantage of machine learning in different fields. We propose a new approach for assessing players’ experience (in particular, their enjoyment) that might mitigate some of the disadvantages of self-report techniques, such as misinterpretation or temporal delay between the experience and reporting during longer experiments.

Based on the assumption that psychophysiological measures, such as heart rate, electrodermal activity, and respiratory activity, correlate with user experience, we suggest combining them with the player’s self-reports to create a machine learning-based model of the player’s enjoyment. A well-trained model will allow game developers to obtain high-quality data about players’ emotions during particular in-game events without questionnaires.

In order to test this approach, we performed a collection of experiments with real participants, trained four different machine learning models, and compared their performances. The results seem to confirm the validity of this technique. We also provide an overview of several types of related psychophysiological measures, along with a very brief biological background and hardware that can be used to collect them. Successfully trained enjoyment models can be used to minimize expenses and accelerate or even partially automate process of evaluating the game experience. Finally, we publish an anonymized version of our dataset to be used for further research on the subject.

## 2. Related Work

Numerous definitions of the term “user experience” have been proposed up to this day [3,4,5,6]. In 2008, Haasenzahl described user experience as an umbrella term used to stimulate research on HCI (human–computer interaction) to focus on aspects that are beyond usability and its task-oriented instrumental values [7]. A survey book focusing on game user experience from 2015 by Bernhaupt lists different definitions of user experience, and it also provides an overview of different evaluation methods applicable to particular game development phases [2]. One of the terms connected closely to digital entertainment and our research is enjoyment. While social research on the motivational appeal of video games is still sparse [8], there is some indication that resolving game tasks and mastering game challenges is closely connected to game enjoyment. In 2011, Tamborini et al. conducted two studies, replicating and extending previous studies [9,10] to provide empirical support for a definition of enjoyment as the satisfaction of both hedonic (arousal, absorption and affect) and nonhedonic (competence, autonomy and relatedness) needs [11].

Kivikangas et al. reviewed many scientific research papers concerning the use of psychophysiological measures in video-games [12]. The review investigated various types of psychophysiological metrics, the pros and cons of their research and particular causes of changes in the subjects’ psychophysiological states. Another study relevant to our research is the work of Drachen et al. who studied correlations between self-reporting and psychophysiological measures [13]. In their research, they used the iGEQ (In-Game Experience Questionnaire) as a self-reporting method in combination with heart rate and electrodermal activity measurement. The In-Game Experience Questionnaire (iGEQ) is a self-report scale for the exploration of the participant’s experience when playing a computer game [14]. Their research indicated that heart rate (HR), as a measure of arousal, is a good correlator with self-report measures of player experience, either positively or negatively. Conversely, they concluded that electrodermal activity (EDA) is only a strong correlator with the negative affect dimension of the iGEQ questionnaire. In 2010, the same authors focused on the effects of a particular game genre (first-person shooter) on the subjects’ physiological states [15]. The importance of game genre within our research is discussed in Section 3. Further research of psychophysiological responses in video-games has focused on studying the effects of various game genres and specific situations on the subject’s psychophysiological states. For instance, Ballard and Wiest studied the effects of violent video-games on the hostility and cardiovascular responses of males [16]. Even though their work contained debatable speculations, they showed that a violent game title (Mortal Kombat) indeed elicited greater cardiovascular reactivity than non-violent games (billiard game). Similar studies were conducted with movies. Codispoti et al. investigated various reactions to emotional movies with an emphasis on the subject’s gender [17]. With a subject sample of 33 females and 27 males, they investigated the cardiac response to highly arousing pleasant and unpleasant films. Based on the correlations found, they concluded that both pleasant and unpleasant movies prompt similar cardiac deceleration. The results of these experiments suggest that there indeed exists a correlation between psychophysiological indicators and the experience and emotions of EE consumers, but the relation to their enjoyment, in particular, is not trivial.

The connection between psychophysiological measures and specific emotions was studied by Mandryk et al., who monitored multiple indicators of subjects playing against computer-controlled opponents as well as other co-located players, as follows:

- EKG (cardiovascular measures)
- HR (heart rate)
- GSR (galvanic skin response)
- EMG (electromyography)
- Resp Rate (respiratory rate)
- RespAmp (respiratory amplitude)

Afterwards, they were able to compile a list of possible correlations between particular psychophysiological measures and subjective responses, as follows [18]:

- Fun significantly correlated with GSR.
- Boredom correlated with EMG.
- Challenge correlated linearly with RespAmp and EMG.
- Ease also correlated linearly with RespAmp and EMG.
- Frustration significantly correlated with GSR and RespRate.

Other publications have addressed the correlation problem of psychophysiological measures with similar results. Drachen, Nacke, Yannanakis and Petersen have produced several papers in the field, indicating how various emotions affect the psychophysiological measures of subjects. In 2009, their results indicated that HR, as a measure of arousal, is a good correlator with self-report measures of player experience, both positive and negative [13,19]. Going deeper into the topic, in 2010, they found that a higher average HR is indicative of players feeling tense and frustrated. In general, a low HR average indicates positive affect, achieving the flow-state, feelings of competence and immersion, and low levels of challenge [15].

Most of the work concerned with modeling focused on game-related events and development processes as opposed to using models for user experience optimization. In 2009, Pedersen et al. investigated the relationship between level design parameters of platform games, individual player characteristics, and experience. They observed correlations between gameplay features (i.e., jumping, running, collected items, killed enemies) and three (commonly studied in context of video-games) emotions: Fun, challenge, and frustration. Their model achieved a performance of 69.18% for fun, 77.77% for challenge, and finally, their best performing model had an accuracy of 88.66% for predicting player frustration [20]. Drachen et al. utilized clustering to model four different types of players in Tomb Raider: Underworld. They used data obtained from 1365 players who completed the game and combined clustering with Emergent Self-Organizing Maps (ESOM). In the end, they were able to assign most of the players (6.37% of players were placed on cluster borders) to one of the four player type clusters: Veterans, Solvers, Runners and Pacifists [21]. Togelius et al. used player modeling as a tool for designing tracks in a race game. Until switching to evolution, they tested and compared using artificial neural networks (ANN) with backpropagation and nearest neighbor classifier unsuccessfully. The evolutionary algorithm was able to design fitting levels for each approximated model of player profile [22]. Most related to our research is the work of Lobel et al. They utilized biofeedback from physiological sensors in real-time for subjects playing the Nevermind game [23]. The game contains flexible elements which are personalized based on data from sensors measuring data such as heart rate, heart rate variability, emotional feedback, and eye movement. For example, in a flooded room, the level of liquid endangering the player rises proportionally to player’s stress level. In 2009, Missura and Gartner proposed an automated difficulty adjustment system based on local player models. They used a combination of player clustering and support vector machines (SVM) to create a dynamic, difficult settings for a simple 2D game, which reportedly improved overall game experience [24].

Since the target of our work was to focus on the autonomic nervous system, we decided not to include facial expression recognition. There has been a fair amount of research done on emotion recognition from facial detection; some of the latest is listed in a review by Latha et al. [25]. Incidentally, the previously mentioned game Nevermind is capable of taking advantage of standard webcams to use emotion-based feeback instead of physiological-based feedback [23]. Chen reviewed different approaches of using audio-visual information for emotional expressions in human–computer interactions [26]. Diaz et al. studied the effects of violent video-games on cognition skills in assessing facial emotions. Their preliminary work suggested that chronic exposure to violent games might have slight effects on the recognition of some emotions (fear, disgust) [27].

Despite numerous studies focusing on using machine learning and physiological measurements in video-games, we have not found any research focused on improving the experience evaluation using these tools. Most of the investigated research is focused on modeling several emotions of players, or on optimizing the development process by modeling the player’s responses on specific game events. Our approach is focused on reducing the costs of the evaluation process as well as optimizing the process by taking advantage of physiological measures produced by the autonomic nervous system (ANS).

## 3. User Experience Evaluation in Video-Games

Nowadays, without using physiology, there is a limited number of methods used for assessing user experience in games: Behavioral observation, interviews, questionnaires, focus groups or heuristic, and game metrics evaluation [2]. These methods (excluding game metrics and heuristic evaluations) come with inherent problems, such as subject bias and interpretation difficulties. For example, while questionnaires provide a quick way to obtain quantitative insights into player feelings and attitudes, they lack the depth of an interview or the objectivity of metrical measures. They also work most reliably when a large number of people are available for testing [2].

We feel that the game development process might benefit from being able to evaluate specific parts of the product (such as game levels or movie scenes) in a more reliable and automated manner. We propose a solution combining the subject’s self-reports with involuntary responses of the autonomic nervous system (ANS), which are impervious to personal bias and provide the data immediately. Similar to the work of Martinez et al. in 2013 [28], our research focuses on using one-dimensional time-series input signals such as an electroencephalograph (EEG), electromyograph (EMG) and galvanic skin response (GSR) for modeling user experience in video-games. Their work utilized blood volume pulse (BVP) and skin conductance (SC) data for training a deep learning neural network model of affect. We propose a similar approach utilizing more physiological indicators and machine learning techniques (including deep learning) to model a single affect (enjoyment). This kind of data is used as an input for a machine learning based model trained to predict the player’s enjoyment.

Our approach relies on the assumption that enjoyment correlates with selected psychophysiological indicators, such as heart rate, electrodermal activity, and respiratory activity. Based on previous research, these measures are closely related to emotional arousal, relaxation, and attention; however, emotions are often considered to be more related to valence-carrying emotions. In 2005, Sherry published a paper explaining links between enjoyment and arousal/relaxation. They stated that emotional stimulation or relaxation can be actively regulated by varying the strength and target of dispositional alignments based on the distance between characters and the self [29]. In this perspective, pleasure and pain as well as arousal and relaxation are neither mutually exclusive nor polar opposites. Instead, enjoyment is seen as relief from overstimulation (through relaxation) or understimulation (through arousal) [30].

The term enjoyment is used as a metric in digital entertainment; however, we left a certain degree of interpretation freedom for subjects when defining the term prior to the experiment. This work was not focused on investigating which of the used indicators affect the particular emotion; we tried solely to model the enjoyment of the subjects during the gameplay. Initially, there were concerns that successful training would not be possible as the measured indicators may not correlate with enjoyment changes; however, these were found to be unsubstantiated after the initial round of experiments described in Section 5.

When evaluating user experience in video-games using automated methods, certain challenges inherent to individual types of games had to be taken into consideration, as follows.

### 3.1. Single-Player

Single-player games provide a clear view of how various game events and situations affect the player’s psychophysiological state (in comparison with multi-player games). The lack of the social aspect of the game makes players less distracted and more focused on the gameplay than when playing with other people. Single-player games provide a straightforward opportunity to use data for the evaluation of individual levels of the game. Unlike in multi-player games, players encounter similar situations throughout the gameplay, which, for example, may provide information on what sections of a particular level are the most (or least) entertaining. In 2011, Hainey et al. discussed motivation differences in single-player and multi-player games [31]. Their study found differences in motivation in single-player and multi-player test subjects stating that competition, cooperation, recognition, and curiosity ranked higher for multi-player and online gamers.

### 3.2. Multi-Player

The multi-player format seems to be more challenging for the user experience evaluation process. Players are set into the same game environment repeatedly; however, the gameplay itself is rarely similar to previous attempts. In addition, different player compositions create a dynamic setting that makes measurements considerably more difficult than in the single-player format.

Furthermore, there are multiple different potentially important influences that could be considered. Game genre, social aspects of the game, players’ sociodemography, or even the combination of all these aspects can have significant effects on players’ responses. For example, while playing a single-player game, a player’s experience is not influenced by the behavior or the performance of their teammates or opponents. In contrast, many multi-player games encourage their players to communicate with their teammates to simulate real-life battlefield experience or to improve their cooperation skills during gameplay. After an initial set of experiments with multi-player games (described in Section 5), we decided to focus on single-player games to prevent any unnecessary interference during later gameplay sessions.

## 4. Psychophysiological Measures

Any research method in which the dependent variable is a physiological measure and the independent variable is behavioral or mental (such as memory) is a psychophysiological method. Physiological measures take many forms and range from blood flow or neural activity in the brain to heart rate variability and eye movements. These measures can provide information about processes including emotion and cognition as well as interactions between them. Physiological measures thus offer a very flexible set of tools for researchers to answer questions about behavior, cognition, and health [32].

All the measures used in our research are parts of the human nervous system, more specifically the autonomic nervous system (ANS), which controls involuntary physiological responses. Measures controlled by the ANS function automatically. Therefore, they can not be affected or controlled consciously, which reduces the risk of unsuccessful experiments or imprecise results. We believe that a combination of traditional techniques with psychophysiological measures will provide a reliable insight into the player experience. Although there is no prior verification of reliability of such a combination, interviews and questionnaires are currently the only way to evaluate the player experience, and therefore the only way to create our model. This section provides a short introduction into the ANS and psychophysiological measures used in our work.

**Figure 1:**
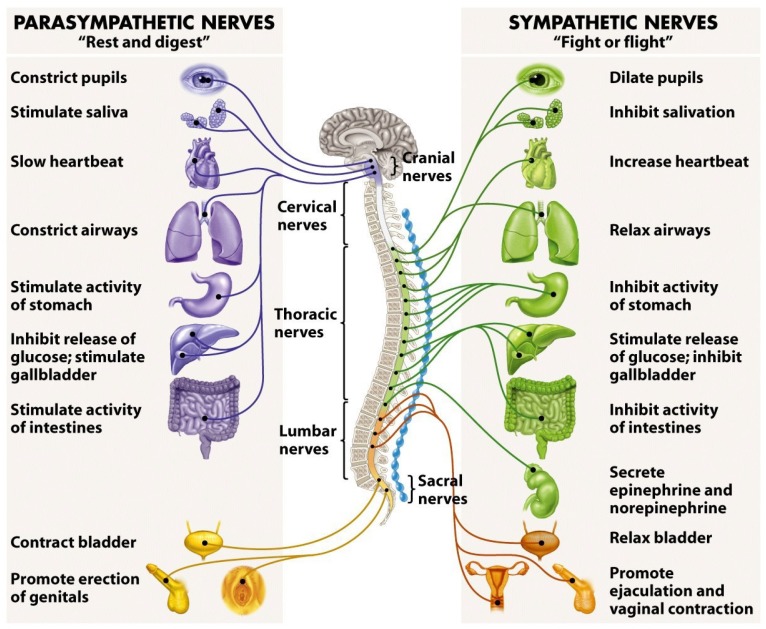
The human autonomic nervous system and its response stimuli [34].

The ANS is comprised of two branches: The sympathetic nervous system (SNS) and the parasympathetic nervous system (PSNS). The easiest way to distinguish differences between these two branches is to associate “fight or flight” responses with the SNS, and “rest and digest” responses with the PSNS. In this manner, the SNS is responsible for “exciting” the body in stressful environments (e.g., competition), and it does so by stimulating the responses shown in Figure 1. More specifically, by stimulating the secretion of adrenaline and noradrenaline, otherwise known as epinephrine and norepinephrine, the SNS prepares the body for stress. More relevantly, the SNS also increases heart rate, the force of contraction, and blood pressure, leading to increased blood flow to the muscles [33]. Therefore, any time the subject feels an “adrenaline rush” before a stressful activity, this is essentially the SNS being stimulated and preparing the subject for competition. In contrast, the PSNS does the opposite and it is responsible for reducing the heart rate and blood pressure in the absence of stress. Essentially, the PSNS helps to facilitate recovery after a stressful event (e.g., competition) by counteracting the effects of the SNS [33]. To put it simply, the SNS increases heart rate and blood flow to the muscles, whilst the PSNS reduces heart rate and peripheral blood flow—in effect, they counteract one another.

### 4.1. Traditional Methods of Psychophysiological State Assessment (Self-Report)

As addressed in Section 3, self-reporting of user experience using traditional methods was used in numerous previous publications. Methods like questionnaires, interviews, narrative techniques, etc. have proven useful for assessing the subject’s experience with the added value of the ability to determine the subject’s feelings and preferences. However, the results of such methods may be biased, especially if subjects are frustrated during the experience due to their inadequate skill level or their emotional state prior to the experiment.

### 4.2. Heart Rate and Heart Rate Variability

Heart rate (HR) is a measure of cardiovascular activity which reflects the emotional state. It has been found to increase for a number of negative emotions (e.g., anger, anxiety, embarrassment, fear, sadness) as well as for some positive emotions (e.g., happiness, joy) and surprise [35].

Heart rate variability (HRV) is the time difference between each successive heartbeat, otherwise known as the R-R interval or the inter-beat interval. The time between each heartbeat is not fixed or consistent, but it varies with every beat—hence the term variability. We used an internal algorithm provided by Zephyr (sensor manufacturer), since the sensor provides HRV automatically without the need to calculate it ourselves. The Zephyr Bioharness sensor used in our experiments provides an internal algorithm that calculates HRV based on ECG data. However, in their validation research, Nepi et al. concluded that the HRV data provided by this sensor is not reliable [36]. This, and the fact that the calculations take place only after the initial five minutes of measured HR, were the reasons that we decided not to use HRV data as an input for our models.

### 4.3. Respiratory Activity

Respiration is measured as the rate of volume at which an individual exchanges air in their lungs. Previous research has found that the respiration rate is increased by emotional arousal and decreased by rest and relaxation [37]. Overall, respiratory activity (RA) is rather easily measured during the experiments. There are several types of device used for measuring respiratory activity, which are worn on the chest, torso, neck, or even the wrist.

### 4.4. Electrodermal Activity

Electrodermal activity (EDA) measures the activity of the eccrine sweat glands and has been found to be a linear correlate to arousal [38]. However, room temperature, humidity, participant activity, and the correct attachment of the electrodes are factors that have to be carefully considered. Phasic EDA is a well researched and valid method to record arousal and has been used for measuring emotions for interaction with systems [39,40]. EDA sensors are typically worn on the fingertips, but nowadays, wrist-worn sensors and even ring sensors are becoming available.

### 4.5. Electroencephalography

Electroencephalography (EEG) provides data about the brain’s electrical activity with millisecond accuracy. Certain features of the signals have been shown to be associated with drowsiness and vigilant attention [41] or to reflect inactivity in the brain regions (smaller use of mental resources [42]). EEG has also been used to study the processing of visual emotional stimuli [43]. However, to date, the use of EEG in game research has been sparse, perhaps due to the complicated nature of the signals, which combined with a complex stimulus, produce a range of methodological challenges [12].

## 5. Experiments

### 5.1. Preliminary Experiments

**Figure 2:**
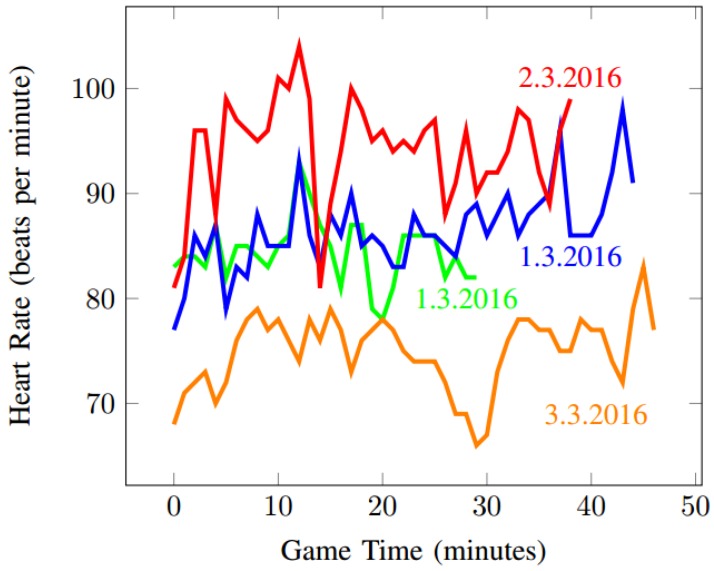
Heart rate during four games of Dota 2, played by the test subject in three sessions.

In the first batch of experiments, we measured only one psychophysiological indicator (heart rate, using the wrist-worn smartwatch sensor Fitbit Surge: https://www.fitbit.com/us/surge) of one participant who played ~20 h of Dota 2 over several days. More information about this batch of experiments can be found in [44] and previous conference paper.) This first set of experiments helped us to support the validity of our hypotheses and establish the process for the subsequent experiments. Figure 2 depicts the heart rate recorded from four games over three different days. Unsurprisingly, we were unable to reliably model player enjoyment using only one psychophysiological indicator.

During the second set of experiments, we expanded the number of measured features to 10 and the number of participants to 31. The measurement details can be found in Table 1 and the following subsections.

### 5.2. Apparatus

For data collection, we used an apparatus consisting of three different sensors and a webcam, as follows:

- Zephyr Bioharness 3 (https://www.zephyranywhere.com/) for measuring heart rate and respiratory and physical activity.
- Neurosky Mindwave Mobile (http://neurosky.com/) for measuring EEG and blinking frequency.
- Grove GSR Sensor (http://wiki.seeedstudio.com/) in combination with Arduino Uno (https://www.arduino.cc/) for measuring electrodermal skin activity.
- Creative Senz3D (https://us.creative.com/) for recording the experiment.

### 5.3. Data Collection

When dealing with psychophysiology, it is important to carefully consider the prior psychophysiological states of the subject. As described in [18] and confirmed by our initial experiments, particular readings of subjects vary depending on several factors. For example, Figure 2 shows the same subject playing the same game four times in three separate sessions over three days. This figure shows that average values of different measures vary significantly based on the subject’s mental and physical state on given day. Psychophysiological measurements can be affected by many variables. Temperature, humidity, attachment of electrodes, individual differences, differences concerning gender, age, time of the day, the use of stimulants, such as coffee or energy drinks, medicaments, etc. can cause different sensor readings [45]. Therefore, we decided to ignore absolute values of HR, RA, EEG, and EDA measurements in the future and use the deviations from the session average instead.

Besides the psychophysiological state, the game format also affects the experimental readings considerably. While in single-player games the players are focused solely on the game experience, in the multi-player format, the social aspect comes into play. To avoid similar interference, we avoided any unnecessary communication with subjects.

**Figure 3:**
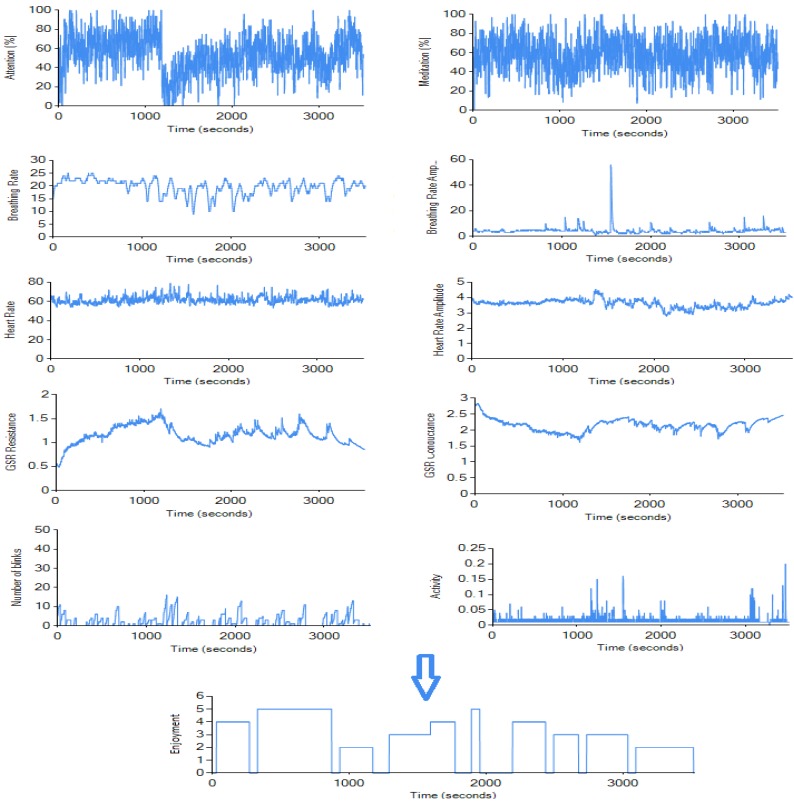
Sample data from our data collection software. Ten features were measured by psychophysiological sensors and one target feature (enjoyment) was collected during a post-game interview.

With the Bioharness sensor, we were not able to access the data in real-time (no real-time API is provided), so the data was processed after the experiments using a simple CSV log parser. Heart rate (250 Hz), heart rate amplitude (250 Hz), breathing rate (100 Hz), breathing rate amplitude (100 Hz), and activity (1 Hz) data were stored in our database. The Mindwave EEG sensor provides the data in real-time, so our database was updated every second during the experiments; attention, meditation, and blinking frequency were stored. Attention and meditation features are calculated on Mindwave Mobile sensor’s chip using NeuroSky’s proprietary algorithm, as described in [46]. The blinking rate was extracted using the sensor’s raw data (512 Hz), detecting repeating anomalies every time an eye-blink happened. These anomalies were caused by movement of the skin on the participant’s forehead. False positives could have been invoked by forcibly frowning or rapid eye movement from side to side (these were fairly rare). The EDA sensor we used was connected to Arduino, and while it was able to send the data in real-time using a serial port, we decided to store it into text files during the experiment and only add it to the database after cleaning the noise. Since the noise in the data was quite rare, we were able to use forward interpolation for filling the missing values in the dataset. As shown in Figure 3, we measured 10 psychophysiological features using three different sensors (listed in Section 5.2). The list of measured features with corresponding units is shown in Table 1.

Afterwards, we calculated the deviations from the session-average values instead of the absolute values for each feature except the blinking frequency. The blinking frequency was represented by three values—the number of times the subject blinked during the last 10, 30, and 60 s. Additionally, we constructed three more proxy features for each measurement to address the temporal structure of the data: the value deviation over the last 10 s, the last 30 s, and the last 60 s (similar to blinking frequency data, we published an anonymized version of our dataset and source code at: https://github.com/Games-and-Simulations/psychophysiological-indicators-data). The final sampling frequency in our database was one measurement per second for all features.

### 5.4. Subject Sample

There were 31 experiment participants with an average age of 28 years. Twenty-six of them were male, and five were female. The youngest of the participants was 20 years old, the oldest was 40, while ∼70% of them were between the ages of 23 and 29. Sixty percent of the participants were either undergraduate or graduate students of computer science. The rest of the participants did not have a significant computer science background. We asked the participants about their experiences with video-games: Nine of them had very limited or no experience with games, meaning that they had tried some games in the past but only had a few hours worth of experience combined in their lifetime. Eleven of the participants identified themselves as gamers, meaning they play video-games regularly. The remaining 11 participants were placed in the “average” group, meaning they play video games occasionally; however, they are not skilled players. Information about their age, gender, and experience with playing video-games was available. However, after performing the feature analysis, this information proved ineffective (or even counterproductive) for training our models and was not used.

### 5.5. Gameplay Session

**Figure 4:**
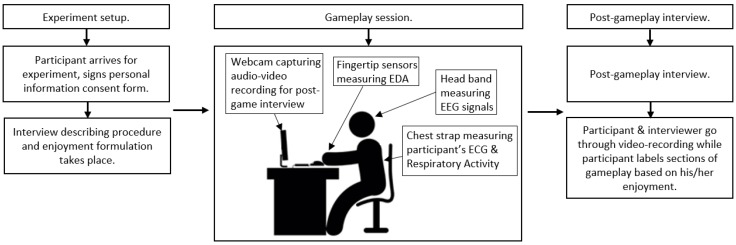
Schema of the experiment procedure.

Prior to the experiment, each participant was informed about the simplified definition of enjoyment based on the definition provided in Section 2. Also, every participant was asked to sign a personal information consent form. Afterwards, it was explained to participants that with the exception of asking for help with controls, dialogue with interviewers was discouraged. Every participant played approximately 45 min of 2–3 different games (in total). The game genres and titles were selected based on each participant’s previous experience with video-games to avoid any discouragement caused by forcing a subject to play inappropriately difficult games. We divided participants into three categories based on their previous experience with video-games. However, not all participants in a category played the same games, e.g., if the subject had already played the game in the past, another game was selected. The transition periods between games were made as fast as possible, and any unnecessary conversations were avoided. After the session, we conducted an interview where participants watched the video recording of their session and labeled their enjoyment level during individual time periods. Enjoyment was measured using a five-item (1 = lowest enjoyment, 5 = highest enjoyment) Likert-type scale adapted from [9]. In games with short levels/segments, we let the participants label the level as one event (for example, Need For Speed: Payback levels are usually 1–3 min long). If the levels/segments were longer, we asked the participants to point out game events that had an effect on their enjoyment levels and labeled between those (e.g., individual fights with enemies and non-fighting periods in Doom). Based on the game and the participant’s ability to assess their enjoyment, the times between enjoyment alterations differed. This assessment is the 11th feature shown in Table 1, labeled as enjoyment, and it served as a target feature for our machine learning models. Figure 4 shows a simple visual schema of the experiment procedure.

## 6. Modeling

We trained four machine learning models to output player’s enjoyment based on measured psychophysiological features. The target values were the enjoyment levels provided by the subjects (on a scale of 1–5, where five represented the highest enjoyment). We tested two different approaches to this problem: Classification and regression. The input for all of our models was a time-series dataset consisting of 39 features: Four features derived for each measure listed in Table 1, except the blinking frequency (three features). The output of our classification models was one of the five classes (enjoyment levels). Our regression models returned a level of enjoyment as a real number.

### 6.1. Methods

We experimented with a feedforward deep neural network, a random decision forest, and a decision jungle methods for our models. The neural network is one of the most common approaches to this kind of task. The random decision forest and decision jungle methods were selected due to their versatility (they work well with both the regression and classification tasks) and implicit feature selection, with no need for scaling and fast training.

#### 6.1.1. Deep Feed Forward Neural Network

A deep neural network is an artificial neural network that has more than one layer of hidden units between its inputs and its outputs [47]. Deep learning methods offer a lot of promise for time series modeling, such as the automatic learning of temporal dependence and the automatic handling of temporal structures like trends and seasonality. Based on the nature of our data, we believed that the deep learning network would be able to successfully model players’ enjoyment during gameplay.

We tested multiple topologies and hyperparameter settings for our neural network model. The model was created in Keras (https://keras.io/) API using Tensorflow (https://www.tensorflow.org/) backend. The best results were obtained using a combination of four hidden layers, each having 128 nodes. We used a rectified linear unit activation function, an *Adam* [48] gradient-based optimization method with an 0.001 learning rate parameter and 20 best features selected by the *SelectKBest* method. We also considered testing the LSTM neural network model. However, after considering that LTSMs do not deal well with gaps in time-series data, we decided against it. As described in Section 5.3, our dataset is not one continuous time series—there are downtimes where target feature (enjoyment) was not labeled.

#### 6.1.2. Random Decision Forest

The decision forest algorithm is an ensemble learning method for classification. The algorithm works by building multiple decision trees and then voting on the most popular output class. Voting is a form of aggregation, in which each tree in a classification decision forest outputs a non-normalized frequency histogram of labels. The aggregation process sums these histograms and normalizes the result to get the “probability” for each label.

Decision trees, in general, are non-parametric models, meaning they support data with varied distributions. For regression tasks, each tree outputs a Gaussian distribution as a prediction. An aggregation is performed over the ensemble of trees to find a Gaussian distribution closest to the combined distribution for all trees in the model [49]. In addition to the listed reasons, their natural ability to overcome overfitting (compared to decision trees) was the reason we chose this method for our task.

For training our decision forest models, we used the Azure Machine Learning Studio framework. Our optimal model had a maximum of 16 decision trees, and the maximum depth of each tree was 32. To build each node of the tree, the maximum number of splits was 256. The number of training samples required to generate a leaf node was two. We used bagging (bootstrap aggregating) as a resampling method.

#### 6.1.3. Random Decision Jungle

A decision jungle consists of an ensemble of a decision directed acyclic graphs (DAGs). By allowing tree branches to merge, a decision DAG typically has a lower memory footprint and a better generalization performance than a decision tree, albeit at the cost of a somewhat higher training time. Similar to decision forests, decision jungles are non-parametric models, which can represent non-linear decision boundaries. They also perform integrated feature selection and classification and are resilient in the presence of noisy features [50].

We used 32 DAGs that could have been created in the ensemble. The maximum depth of the graphs to create the ensemble was 64, with a maximum width of 256. The number of optimization steps per decision DAG layer was 16,000. As with the decision forests, bagging (bootstrap aggregating) was used as a resampling method.

### 6.2. Feature Extraction

Prior to training the models, we filtered the noise in our data by replacing missing values, created several proxy features more suited as inputs for our model, and conducted typical data pre-processing procedures. During the experiments, 10 features were saved into our database: Heart rate, heart rate amplitude, breathing rate, breathing rate amplitude, activity, attention, meditation, blinking rate, gsr_conductance, and gsr_resistance. Additionally, information about the subjects and their IDs were also saved; however, they were not used in the training process. In the end, we ended up with 39 features as input for our models. The extraction process is explained in subsections below.

#### 6.2.1. Replacing Missing Values

**Figure 5:**
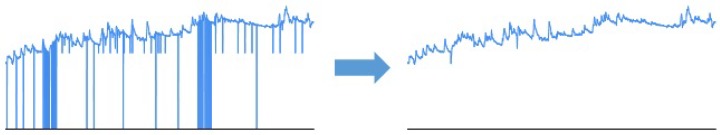
Illustration of forward interpolation applied to data provided by an electrodermal activity (EDA) sensor.

Out of three sensors used, one of them provided occasional noise in the data which needed to be filtered. We used forward interpolation, replacing the missing value with the previous one from the dataset. This process was possible since the noise samples were not clumped together (no two consecutive samples were missing). An illustration of this process is shown in Figure 5.

#### 6.2.2. Adding Derived Features

As explained above, if the absolute values provided by our sensors were used, our models would be vulnerable to prior physiological states of subjects (amount of sleep, caffeine, etc.), affecting the results. Therefore, we used the average value for every measurement from each experiment session to calculate deviation values for our dataset. We calculated the average value for each feature (except the blinking rate, which was already represented in a similar fasion) throughout the experiment session. Afterwards, we subtracted each raw value from the sensors from the average value and stored is as the deviation, e.g., if the heart rate of the subject was 85 and the average heart rate in their session was 75, we stored the value 10 as the deviation.

In addition to adding deviation features, we constructed derived features representing the deltas (deviations) of each feature within particular time windows: 10, 30 and 60 s. This means that we created three additional rows for each feature, representing how much the value of each feature changed in a particular time window. This was done based on belief that the subjects do not respond to gameplay changes and events instantaneously, and it has proven difficult to determine how much time it takes for a particular physiological measure to change.

**Figure 6:**
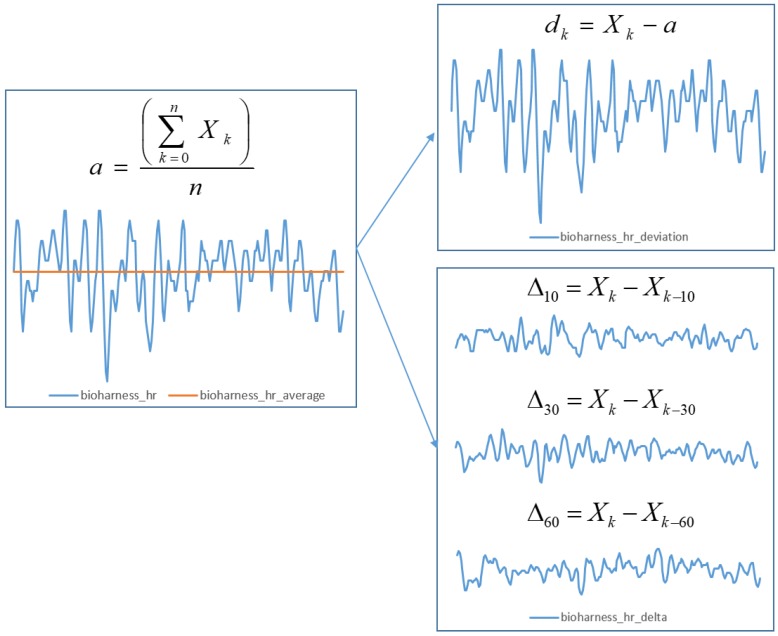
Illustration of feature extraction process, including the derivation of additional features based on raw physiological sensoric data. After calculating the session average for each measurement, we derived a value of deviation from the average as well as delta values (change of each feature over three different time windows). The X-axis represents the sample number (time) and the Y-axis represents the value of the feature.

After this process, we used four different values for each feature—deviation and the deviation in the last 10, 30, and 60 s—and did not use the average and raw values. Figure 6 shows an illustration of the feature extraction process.

#### 6.2.3. Ignoring Intervals with Missing Enjoyment Label

Since enjoyment was used as a target value, we did not include the intervals where it was missing in our training. Enjoyment was labeled on an integer 1–5 scale during the experiments, and unlabeled values were assigned zero values and were not used in further training. These intervals occurred when no actual gameplay took place: Loading times, switching games, explaining controls, etc.

#### 6.2.4. Normalization

Finally, each feature was scaled to the range of [−1, 1]. This applied only to our neural network model, since random forests and jungles deal with unscaled data without problems.

### 6.3. Feature Selection and Cross-Validation

For the neural network model, we applied a feature selection algorithm to determine the optimal subset of relevant features. We used the univariate linear regression tests method in combination with *SelectKBest* method provided by the scikit-learn (https://scikit-learn.org/) library. After attempting to train our model using different numbers of features, we observed that it is not feasible to successfully train the model using less than 10 features. Later, we decided to use 20 out of 39 features to train our model (Out of eight features provided by Mindwave sensor, only one (meditation deviation from average) was selected for training. Features related to breathing rate were prioritized over heart rate-related features (five vs. three). All features provided by our GSR sensor were selected.). Using more than 20 features did not improve the results enough to compensate for the increase in training time.

Since the random forest and random jungle algorithms perform feature selection inherently, we did not replicate the feature selection process for these models.

As our dataset was relatively small, we used 10-fold cross validation for each model to prevent overfitting. None of the tested models showed more than 2% disparity in all folds. All the results (error/accuracy of trained models) presented in this paper represent an average value over all of the folds.

### 6.4. Results

The goal of our research was to create a ready-to-use model which can reliably predict players’ enjoyment during gameplay. The enjoyment values gathered during the experiments had integer values on a scale from 1–5. The input to our models was 39 features extracted from psychophysiological sensoric data, and the output was the key feature enjoyment. When investigating as a regression problem, our models output a real number which should have been as close to measured enjoyment value as possible. When treating as a classification problem, we took all the inputs of the model and classified each sample into one of five classes (enjoyment scale). The detailed results are portrayed further.

After feature extracion and pre-processing of the data, 75–177 samples remained, where each sample represented one second of labeled gameplay, with a total of ∼21 h of gameplay time. The data was divided into a training test and test set using the 80%/20% split for each model.

When training the regression models, our goal was to minimize the root mean squared error (RMSE) values, which represent the deviation between the model output and the enjoyment level indicated by the test subjects.

Our neural network regression model produced a real number representing the predicted value of enjoyment. Table 2 lists results of different tested settings and topologies of the neural network.

**Figure 7:**
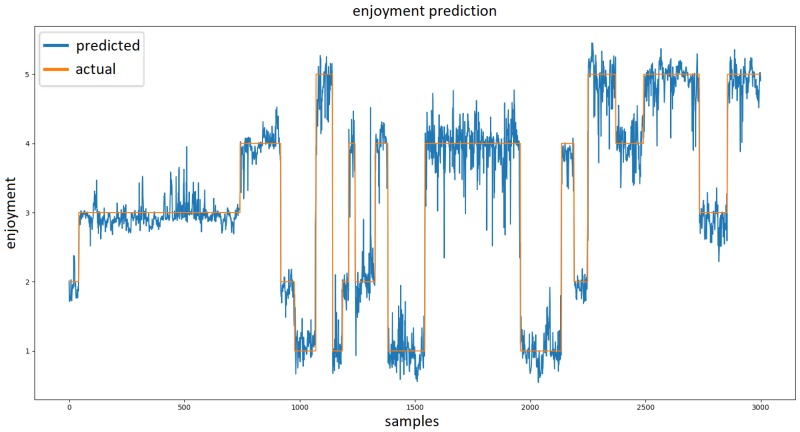
Visualization of the output of our neural network regression model. The orange plot represents the actual reported enjoyment values while the blue plot represents enjoyment predicted by the model.

**Figure 8:**
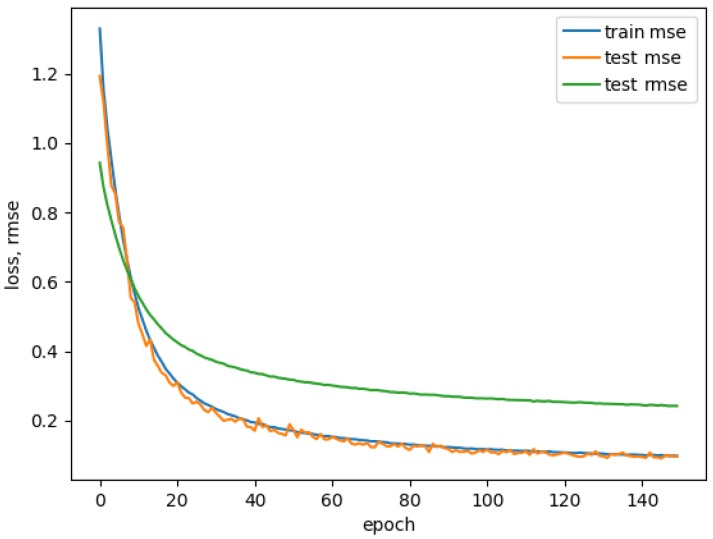
Mean squared error (MSE) on training and testing dataset, and root mean squared error (RMSE) on testing dataset of our neural network model.

Based on the topology, the training process took from 30 min to a maximum of nine hours (with 10-fold cross validation). Figure 7 show how real values produced by model compare to labeled enjoyment during a portion of single experiment. The best performing model produced an RMSE of 0.3157 (see Figure 8). The performance of this model is shown in Figure 7 and Figure 8.

The random decision forest model for both the regression and classification problems was trained using Azure Machine Learning Studio (AMLS): https://studio.azureml.net/. This framework allows for quick implementation of different topologies and easy parameter changes. Also, online training of different models without needing to worry about computing power has proven useful for our work. We used AMLS for training three models: Random decision forest regression, random decision forest classification, and random decision jungle classification. As previously stated, no feature selection was required, since these methods provide inherent feature selection. We provided a pre-processed (scaled data without rows where no enjoyment value was present) CSV file with measured data to the models and used the same 80%/20% train/test split as with our neural network model.

The random decision forest regression model had an RMSE of 0.1991. Both of these results can be considered satisfactory. The regression and classification performance results for the random forest model with different tested settings are listed in Table 3.

**Figure 9:**
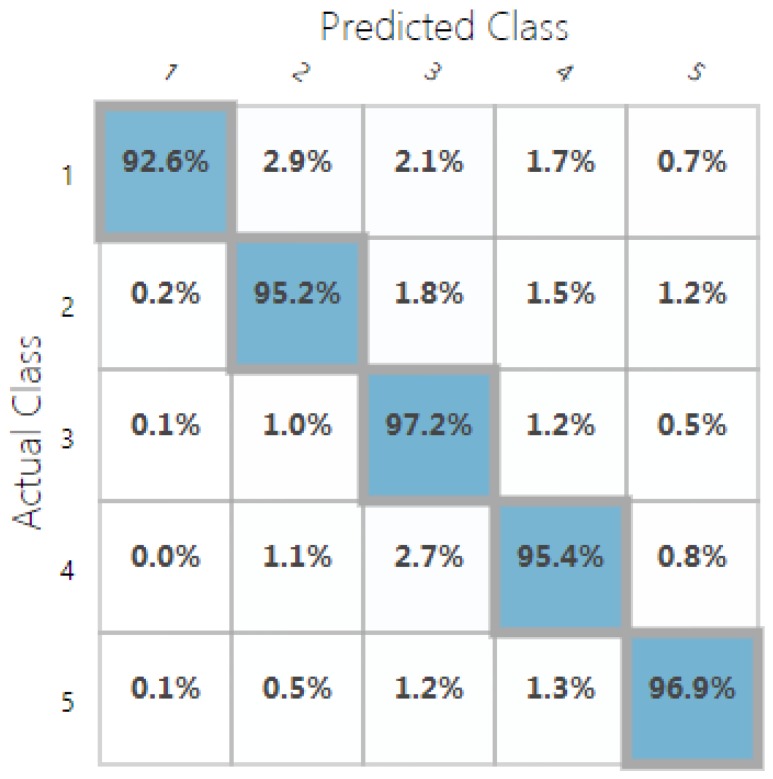
Confusion matrix of the trained random decision forest classification model.

**Figure 10:**
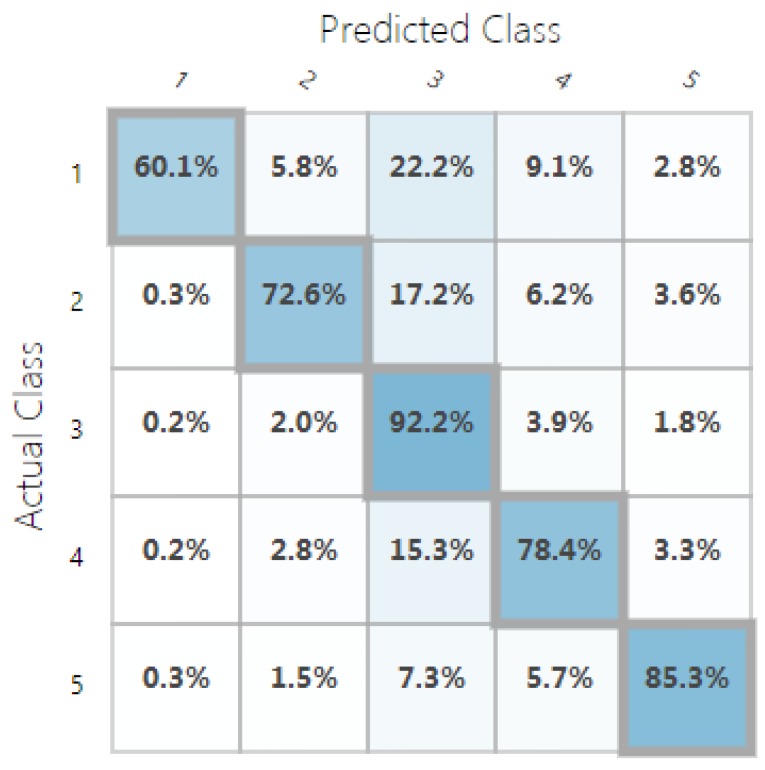
Confusion matrix of the trained random decision jungle classification model.

For classification, we used the accuracy metric defined as the percentage of the correctly predicted samples (using 5 possible classes). Our random decision forest classification model was trained with ∼96% accuracy, and the random decision jungle model performed with ∼84% accuracy. Confusion matrices in Figure 9 and Figure 10 show the predicted vs. observed values for all the five classes. The different settings and topologies of the random decision forest and jungle models are showed in Table 3 and Table 4.

## 7. Conclusions & Future Work

This paper aimed to simplify and automate the process of evaluating the user experience in digital entertainment (especially video-games) and to mitigate some of the drawbacks of self-reporting methodology. We proposed an alternative approach: using psychophysiological measures, such as heart rate, electrodermal activity or respiratory activity in combination with machine learning methods.

We provided an introductory analysis of various available physiological data sources, their relevance to user experience modeling, and technical prerequisites for their collection. Several drawbacks using psychophysiological data were found during our research, such as differences in the prior physiological states of the subjects and their negative effects on experiments. Afterwards, we demonstrated the process of gathering real-time data produced by the autonomic nervous system and involuntary physiological responses and showed how they can be post-processed and used to train machine learning models.

Four different machine learning models were successfully trained using different parameters for optimization. These models were able to reliably predict the enjoyment of players during a video-game session based on their psychophysiological data. Data fed to these models consisted of 20–39 features measured during approximately hour-long gameplay sessions of 31 subjects, totaling ∼70,000 samples. Based on the good performance of these models, as shown in Table 5, we conclude that machine learning and psychophysiological measures can be useful additions or even alternatives to typical means of assessing user experience in the digital entertainment business. These models can prove as useful in larger sample sizes for evaluating user experience in digital entertainment, especially with well-built testing infrastructure. Even though the proposed approach does not entirely escape the disadvantages of self-reporting techniques for measuring user experience, with a sufficient number of participants, it can definitely be useful as an optimization tool for replacing questionnaires and interviews with a successfully trained machine learning model.

For future work, we plan to gather more experimental data (with a more diverse subject sample) and test more machine learning methods to train the enjoyment model. We also hope to set up a testing environment for real-time enjoyment evaluation using trained models and apply this into a real development process.

## Figures and Tables

**Table 1:** List of measured psychophysiological features and sensors used.

Sensor	Measured Feature	Data Range	Unit
Zephyr Bioharness 3	Heart Rate (HR)	25–240	BPM (beats per minute)
Zephyr Bioharness 3	Heart Rate Amplitude (HRAmp)	0.25–15	mV
Zephyr Bioharness 3	Breathing Rate (BR)	4–70	BPM (breaths per minute)
Zephyr Bioharness 3	Breathing Rate Amplitude (BRAmp)	0–65,534	16 bit unsigned number
Zephyr Bioharness 3	Activity	±16 (any axis)	VMU (vector magnitude in g)
Seeedstudio Grove GSR Skin Sensor Module	GSR Conductance (GSRCon)	0–1023	Siemens
Seeedstudio Grove GSR Skin Sensor Module	GSR Resistance (GSRRes) (1/Conductance)	0–1023	Ohm
Neurosky Mindwave Mobile	Attention	0–100	%
Neurosky Mindwave Mobile	Meditation	0–100	%
Neurosky Mindwave Mobile	Blinking Frequency (BF)	0–65,534	16 bit unsigned number

**Table 2:** Detailed regression results of the Deep Feed Forward Neural Network model. Each tested topology is characterized by a specific configuration of layers and units per layer (see the first table column).

Network Topology	Learning Rate	# of Features	RMSE
64, 32	0.01	10	1.3122
64, 32	0.01	15	1.1147
64, 32	0.01	20	0.9813
64, 32	0.001	10	1.1605
64, 32	0.001	15	1.0503
64, 32	0.001	20	0.9737
64, 64, 32	0.01	15	0.8587
64, 64, 32	0.001	15	0.7982
64, 64, 32	0.01	20	0.8101
64, 64, 32	0.001	20	0.7971
64, 64, 64, 64	0.01	15	0.6691
64, 64, 64, 64	0.001	15	0.6821
64, 64, 64, 64	0.01	20	0.6584
64, 64, 64, 64	0.001	20	0.6191
128, 128, 128, 128	0.01	15	0.4979
128, 128, 128, 128	0.001	15	0.4421
128, 128, 128, 128	0.01	20	0.4612
128, 128, 128, 128	0.001	20	0.3157

**Table 3:** Detailed regression and classification results of the Random Decision Forest models. Each model uses a different number of trees, maximum depth, and minimal sample split (first three columns).

# of Trees	Max Depth	Min Samples Split	RMSE	Accuracy
8	8	128	1.0181	54.14%
8	16	128	0.6876	89.51%
8	16	256	0.6545	90.04%
16	8	128	0.7541	70.78%
16	16	128	0.4611	89.97%
16	16	256	0.3348	92.29%
16	32	128	0.2517	95.67%
16	32	256	0.1991	96.04%
32	16	128	0.3422	93.88%
32	32	256	0.3022	95.56%

**Table 4:** Detailed classification results of the Random Decision Jungle model. DAG: Decision directed acyclic graph.

# of DAGs	Max Depth	Max Width of DAGs	Optimization Steps	Accuracy
8	32	128	2048	59.77%
8	64	128	2048	62.48%
8	64	256	2048	64.72%
16	32	128	4096	65.57%
16	32	128	8000	70.04%
16	64	256	8000	78.26%
32	32	128	8000	71.68%
32	32	128	16,000	81.53%
32	64	256	16,000	84.05%

**Table 5:** Comparison of trained machine learning models.

Machine Learning Method	Problem Approach	Score
Feed forward neural network	Regression	RMSE: 0.3157
Random decision forest	Regression	RMSE: 0.1991
Random decision forest	Classification	Acc.: 0.9604
Random decision jungle	Classification	Acc.: 0.8405
